# The Long-Term Neuroprotective Effect of the Endocannabinoid 2-AG and Modulation of the SGZ’s Neurogenic Response after Neonatal Hypoxia-Ischemia

**DOI:** 10.3390/pharmaceutics15061667

**Published:** 2023-06-07

**Authors:** Gorane Beldarrain, Enrique Hilario, Idoia Lara-Celador, Marc Chillida, Ana Catalan, Antonia Ángeles Álvarez-Diaz, Daniel Alonso-Alconada

**Affiliations:** 1 Department of Cell Biology and Histology, School of Medicine and Nursing, University of the Basque Country (UPV/EHU), 48940 Leioa, Spain; 2 Psychiatry Department, OSI Bilbao-Basurto, Basurto University Hospital, 48013 Bilbao, Spain; 3 Neuroscience Department, University of the Basque Country (UPV/EHU), 48013 Leioa, Spain; 4 Biobizkaia Health Research Institute, 48903 Barakaldo, Spain; 5 CIBERSAM, Centro Investigación Biomédica en Red de Salud Mental, 28007 Madrid, Spain; 6 Department of Psychosis Studies, Institute of Psychiatry, Psychology and Neuroscience, King’s College London, London SE5 8AF, UK

**Keywords:** neonatal hypoxia-ischemia, endocannabinoid system, 2-AG, neuroprotection, neurogenesis

## Abstract

Neonatal hypoxia-ischemia (HI) often causes hypoxic-ischemic encephalopathy (HIE), a neurological condition that can lead to overall disability in newborns. The only treatment available for affected neonates is therapeutic hypothermia; however, cooling is not always effective to prevent the deleterious effects of HI, so compounds such as cannabinoids are currently under research as new therapies. Modulating the endocannabinoid system (ECS) may reduce brain damage and/or stimulate cell proliferation at the neurogenic niches. Further, the long-term effects of cannabinoid treatment are not so clear. Here, we studied the middle- and long-term effects of 2-AG, the most abundant endocannabinoid in the perinatal period after HI in neonatal rats. At middle-term (postnatal day 14), 2-AG reduced brain injury and increased SGZ’s cell proliferation and the number of neuroblasts. At post-natal day 90, the treatment with the endocannabinoid showed global and local protection, suggesting long-lasting neuroprotective effects of 2-AG after neonatal HI in rats.

## 1. Introduction

Hypoxic-ischemic encephalopathy (HIE) caused by neonatal hypoxia-ischemia (HI) affects 2–3/1000 newborns in developed countries [1,2] and around 10–20/1000 babies in developing ones [3]. HIE represents a great contribution to overall disability worldwide [2] and yet, the only therapy that has been approved is therapeutic hypothermia. Therapeutic hypothermia has proven to reduce the risk of death and neurodevelopmental disability; however, the efficacy of this treatment is highly dependent on the timing of initiation, as well as on the depth and duration of the insult [4], which makes the search for new alternative therapies a priority.

The modulation of the endocannabinoid system (ECS), an important regulator of a plethora of physiological brain functions, has revealed neuroprotective potential against brain injury, as it targets deleterious events such as glutamate excitotoxicity, oxidative stress, and inflammation [5,6,7]. In fact, the exogenous administration of cannabinoids [8,9,10,11] or the inhibition of endocannabinoid degradation [12], have been revealed as potential neuroprotective strategies for the treatment of HIE in different preclinical models [5].

Together with neuroprotection, augmented neurogenesis may ameliorate brain injury after HI. The ability to generate new neurons and glial cells remains postnatally in two areas, the subventricular zone of the lateral ventricles and the subgranular zone (SGZ) of the dentate gyrus (DG) of the hippocampus [13,14,15]. These neurogenic niches contribute to the plasticity of the newborn brain in physiological conditions [16,17,18,19], but the global damage induced by HI may affect these areas and also their proliferative/neurogenic capacity [20]. As the ECS participates in brain development-related processes such as cell proliferation, differentiation, and survival during gestation and after childbirth [21], compounds that modulate the ECS may be promising neurogenic agents for the treatment of neonatal brain injury.

Discovered 30 years ago, the endogenous cannabinoids (or endocannabinoids) *N*-arachidonoylethanolamine (anandamide) and 2-arachidonoyl-*sn*-glycerol (2-AG) are the main activators of the ECS [22,23,24,25]. 2-AG is the prevalent endocannabinoid in the perinatal period [7], and we wondered if its exogenous administration after perinatal asphyxia could enhance long-lasting neuroprotection and/or modulate the proliferative/neurogenic capacity of the neonatal brain.

Thus, the main objectives of this work were (i) to evaluate the neuroprotective potential of 2-AG at both medium- and long-term and (ii) to analyze the capacity of the endocannabinoid to modulate SGZ’s neurogenic niche cell proliferation and neurogenesis after HI in neonatal rats.

## 2. Results

### 2.1. Body and Brain Weight

On the day of the hypoxic-ischemic procedure (P7), there were no differences in the body weight of animals. Seven days later (P14), the body weight of non-treated HI animals was 23.65 ± 3.47 g, significantly lower than sham animals (28.33 ± 1.33 g; ** *p* < 0.01) and those treated with 2-AG (27.39 ± 2.70 g; ## *p* < 0.01). Consistent with this data, the brain’s weight of non-treated HI animals (0.93 ± 0.11 g) was also smaller (*p* < 0.0001) than that of sham (1.19 ± 0.5; **** *p* < 0.0001) and 2-AG treated ones (1.15 ± 0.08 g; #### *p* < 0.0001). The results are shown in Table 1.

### 2.2. Evaluation of Hemispheric and Hippocampal Injury at P14

The rat model of neonatal hypoxia-ischemia often generates cortical, subcortical, and/or hippocampal tissue loss over time, which will in turn develop into a decrease in the ipsilateral (damaged)/contralateral (non-damaged) ratios of hemispheric areas. Contralateral hemispheres do not suffer brain damage, serving as internal controls.

**Figure 1:**
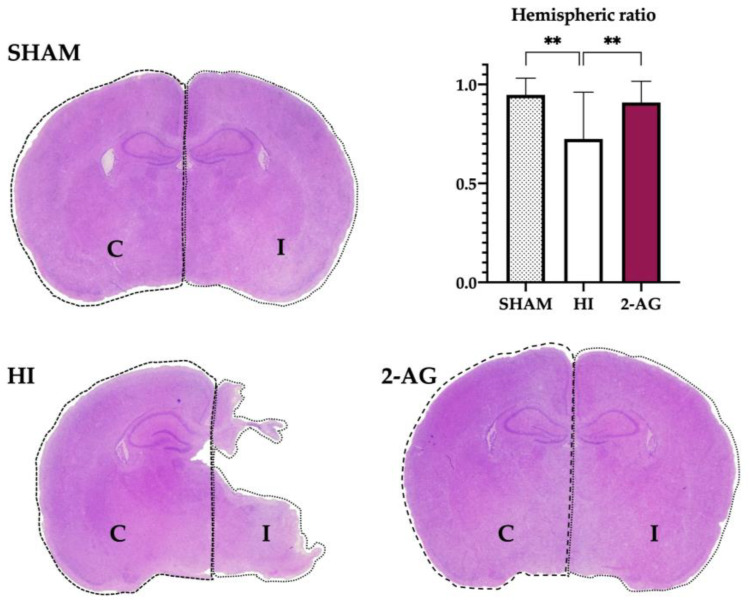
2-AG treatment helps revert the evident damage caused by HI on the ipsilateral hemisphere at P14. Representative brain slices at the level of mid-dorsal hippocampus and thalamus (Bregma −1.80 mm) of sham, non-treated HI, and 2-AG-treated animals. The graph shows the hemispheric ratios of each group at P14. Non-treated HI animals (HI) show evident damage signs in the ipsilateral hemisphere, which can also be observed in the lower hemispheric ratio values of the graph. When comparing the hemispheric ratios of HI and 2AG groups, significant differences were found (** *p* < 0.01). The ipsilateral hemisphere of 2-AG treated animals (2-AG) shows no signs of evident damage and hemispheric ratio values are similar to sham.

At P14, regional cross-sectional brain area measurements at the level of dorsal hippocampus and thalamus from non-treated HI animals showed substantial damage/infarction in the ipsilateral hemisphere compared to sham animals, with hemispheric ratios of 0.72 ± 0.24 mm^2^ for HI and 0.95 ± 0.08 for sham. In 2-AG treated rat pups, the signs of infarction reduced significantly (*p* < 0.01) when compared to non-treated HI animals, with hemispheric ratios of 0.91 ± 0.11 mm^2^, similar values to those observed for sham (Figure 1).

**Figure 2:**
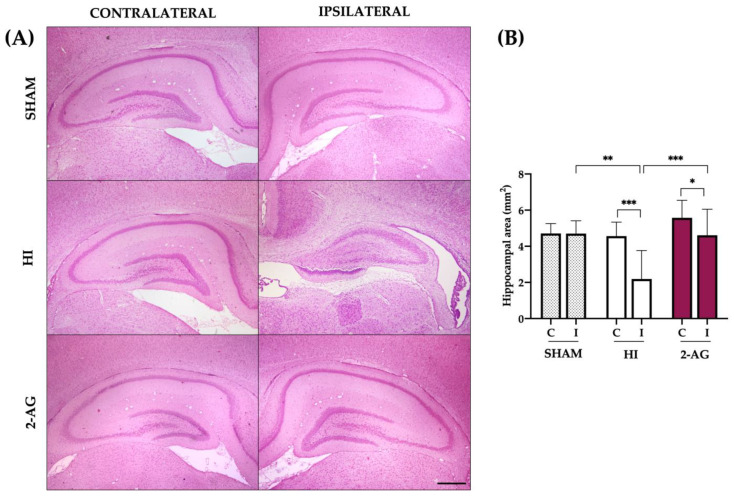
Hippocampal area reduction after HI is reverted with 2-AG treatment at P14. (**A**) Representative microphotographs of the contralateral and ipsilateral hippocampi from sham, HI, and 2-AG animals. Microphotographs were taken at 4× magnification (Scale bar: 500 μm). (**B**) The hippocampal area was significantly smaller on the ipsilateral side of the HI animals compared to the contralateral side (*** *p* < 0.001) and also to sham-I (** *p* < 0.01). The ipsilateral hippocampi of 2-AG treated animals were smaller (* *p* < 0.05) than the contralateral ones. This effect of HI was reverted after 2-AG treatment, as the hippocampal area in the ipsilateral hemisphere of 2-AG animals was significantly bigger (*** *p* < 0.001) than in HI animals and similar to sham-I.

The measurement of the areas of the hippocampi (Figure 2) also revealed evident ipsilateral injury seven days after HI (P14). Sham animals showed no differences between contralateral (4.71 ± 0.54 mm^2^) and ipsilateral (4.70 ± 0.70 mm^2^) hippocampal areas. In non-treated HI animals, the area of the contralateral hippocampi was 4.57 ± 0.77 mm^2^, significantly bigger (*p* < 0.001) than in the ipsilateral ones, which was 2.19 ± 1.57 mm^2^. In 2-AG treated animals, the area of the contralateral hippocampi (5.58 ± 0.97 mm^2^) was significantly bigger (*p* < 0.05) than in the ipsilateral ones (4.61 ± 1.44 mm^2^).

When comparing groups, HI reduced the area of the ipsilateral hippocampal (sham vs. HI: *p* < 0.01). Noteworthy, 2-AG-treated animals revealed hippocampal protection at P14, as this group showed a significantly bigger (*p* < 0.001) ipsilateral hippocampal area when compared to non-treated HI rat pups, with similar values than those observed for sham (Figure 2).

### 2.3. Neuropathological Score at P14

**Figure 3:**
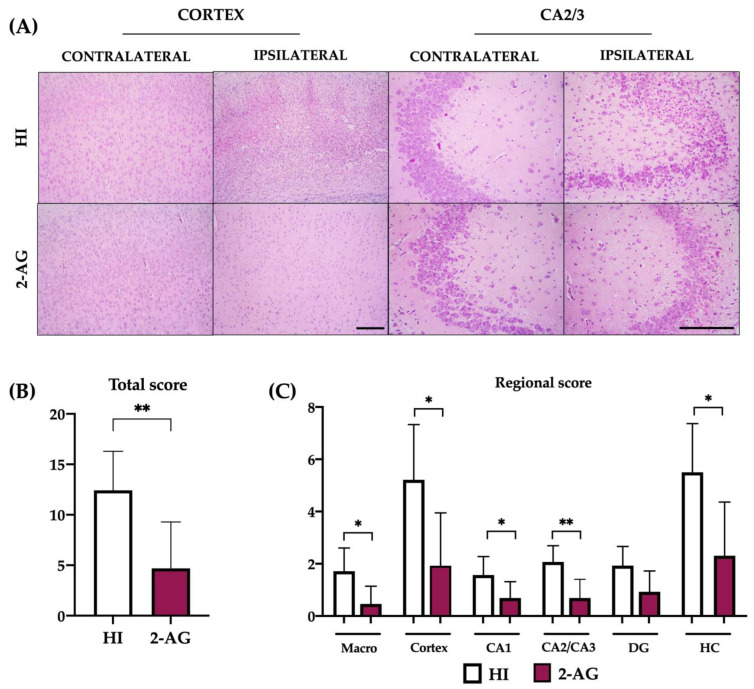
Treating animals with 2-AG results in lower neuropathological score values at P14. (**A**) Representative microphotographs of the contralateral and ipsilateral cortex and CA2/3 regions of non-treated HI and 2-AG treated animals. Cortex images were taken at 10× magnification (Scale bar: 200 μm). CA2/3 images were taken at 20× magnification (Scale bar: 100 μm). (**B**) Global neuropathological score revealed significant (** *p* < 0.01) reduced damage in 2-AG-treated rat pups. (**C**) Regional neuropathological scores were performed macroscopically (macro), in the cortex, in the CA1, CA2/3, and dentate gyrus (DG) areas of the hippocampus, and in the whole hippocampus (HC). Scores were higher in non-treated HI animals than in 2-AG treated animals in all regions, but differences were only significant in the cortex (* *p* < 0.05), CA2/3 region (** *p* < 0.01), and whole hippocampus.

We next used a neuropathological score (Figure 3) to further assess the possible protective effect of the endocannabinoid 2-AG in comparison with non-treated pups. The highest punctuation (and highest damage) for the global score was 21 points, obtained as the sum of the regional scores (see Section 4).

As shown in Figure 3B, the global score for HI animals was 12.43, whereas 2-AG-treated rats obtained a significant (*p* < 0.01) lower neuropathological score of 4.69. The regional score assessment revealed that, when compared to non-treated HI animals, pups receiving 2-AG had significantly lower neuropathological values in the macroscopical injury (*p* < 0.05; HI: 1.71 vs. 2-AG: 0.46), cortex (*p* < 0.05; HI: 5.21 vs. 2-AG: 1.92), CA1 (*p* < 0.05; HI: 1.57 vs. 2-AG: 0.69) CA2/3 (*p* < 0.01; HI: 2.07 vs. 2-AG: 0.69), and total hippocampus (*p* < 0.05; HI: 5.50 vs. 2-AG: 2.30) (Figure 3C).

### 2.4. Cellularity of the DG at P14

After evaluating the histopathological characteristics of the hippocampus from both treated and non-treated HI animals, we next focused on the hippocampal DG, where the SGZ is located. 

**Figure 4:**
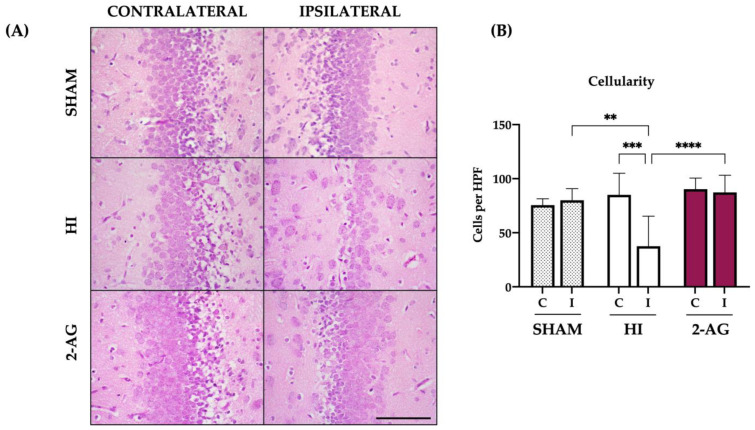
2-AG-treated animals show higher cellularity in the DG of the hippocampus at P14. (**A**) Representative microphotographs of the DG of the hippocampus from sham, non-treated HI, and 2-AG-treated animals. Images were taken at 40× magnification (Scale bar: 100 μm) (**B**) Sham animals showed no significant differences in cellularity of the DG from contralateral and ipsilateral hemispheres. On the contrary, the ipsilateral DG of the HI group showed reduced cellularity compared to the contralateral one (*** *p* < 0.001). Cellularity of the DG in 2-AG-treated animals did not show any significant differences. When comparing ipsilateral DGs of the three groups, significant differences were found between sham and HI animals (** *p* < 0.01) and also between non-treated HI animals and the ones who received 2-AG treatment (**** *p* < 0.0001).

Sham animals showed no significant differences in the cellularity of DG, with 75 ± 5 cells per high power field in the contralateral DG and 80 ± 10 cells in the ipsilateral DG. In non-treated HI animals, we observed reduced cellularity (*p* < 0.001) in the ipsilateral DG: the total number of cells of the contralateral DG was 85 ± 20, whereas the ipsilateral DG showed 37 ± 27 cells per high power field. On the contrary, the cellularity in 2-AG treated animals was similar for both contralateral and ipsilateral DGs, with 90 ± 10 and 87 ± 15 cells per high-power field, respectively. Indeed, the ipsilateral DG cellularity from non-treated pups also showed differences between groups, with higher cellularity values in the ipsilateral DGs of both sham (*p* < 0.01) and 2-AG treated animals (*p* < 0.0001) when compared with that from non-treated pups (Figure 4). 

### 2.5. Ki67 Cell Counts in SGZ and Whole Hippocampus

**Figure 5:**
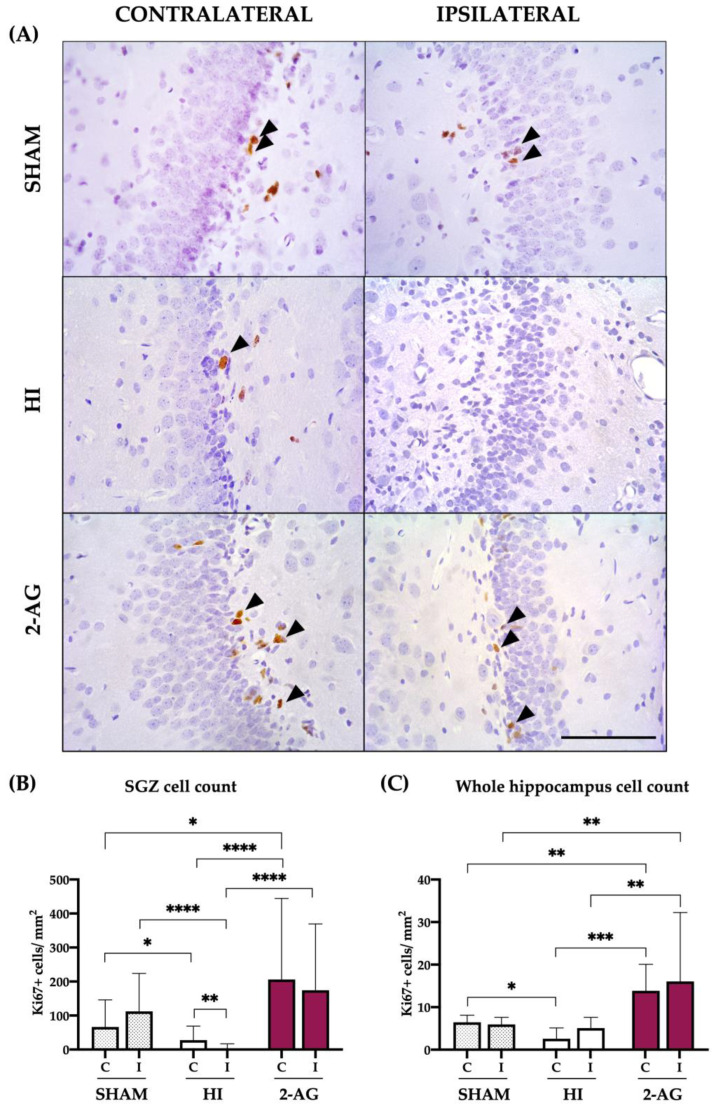
Ki67-positive cells in the SGZ and whole hippocampus after HI and subsequent 2-AG treatment at P14. (**A**) Representative microphotographs of Ki67+ cells in the contralateral and ipsilateral SGZs from sham, HI non-treated, and 2-AG-treated rat pups. Positive cells are identified with black arrows. Images were taken at 40× magnification (Scale bar: 100 μm). (**B**) In the SGZ, HI induced a decrease in the number of Ki67+ cells in the ipsilateral hippocampi (** *p* < 0.01 vs. HI-C; **** *p* < 0.0001 vs. sham-I). Contralateral counts from HI animals also showed reduced proliferation after HI (* *p* < 0.05 vs. sham-C). 2AG-treated animals showed higher Ki67 cell counts in contralateral SGZ compared to sham (* *p* < 0.05) and HI (**** *p* < 0.0001). Importantly, 2AG showed higher Ki67 cell counts in the ipsilateral SGZ (**** *p* < 0.0001) when compared to non-treated animals. (**C**) In the whole hippocampus, HI reduced cell proliferation in the contralateral side (* *p* < 0.05 vs. sham). 2AG-treated animals revealed higher Ki67 cell counts in both contralateral (** *p* < 0.01 vs. sham-C; *** *p* < 0.001 vs. HI-C) and ipsilateral (** *p* < 0.01 vs. sham-I; ** *p* < 0.01 vs. HI-I) hippocampi.

We used Ki67 to study cell proliferation in the SGZ and whole hippocampus of P14 rats (Figure 5). We first analyzed the possible differences between the contralateral and ipsilateral SGZs from each group, to continue with the inter-group analysis.

Results shown in Figure 5A reveal that the contralateral and ipsilateral SGZs from sham animals showed 66.34 ± 79.54 and 112.2 ± 111.8 Ki67 positive cells per mm^2^, with no statistical differences between them. Non-treated HI animals showed low Ki67 positive cell counts, with the contralateral SGZ of those animals showing higher (*p* < 0.01) cell counts when compared to the ipsilateral: 27.88 ± 40.98 vs. 3.5 ± 13.53 Ki67 positive cells per mm^2^. In 2-AG-treated animals, the contralateral SGZ revealed 206.2 ± 238.2 Ki67 positive cells per mm^2^, whereas the ipsilateral SGZ showed 174.5 ± 194.9 cells per mm^2^, with no significant differences between hemispheres. 

When comparing sham and HI experimental groups, HI induced a decrease in the number of Ki67 positive cells in both SGZs, being more pronounced in the ipsilateral one (*p* < 0.05: Sham-C vs. HI-C; *p* < 0.0001: Sham-I vs. HI-I). Interestingly, Ki67 positive cell counts in animals treated with 2-AG turned out to be significantly higher both in the contralateral (*p* < 0.0001) and the ipsilateral (*p* < 0.0001) SGZs when compared to non-treated HI animals.

We also evaluated Ki67 expression in the whole hippocampus (Figure 5B). The contralateral and ipsilateral hippocampi from sham animals showed 6.44 ± 1.67 and 5.95 ± 1.66 Ki67 positive cells per mm^2^, with no statistical differences between them. In non-treated HI pups, contralateral and ipsilateral hippocampi showed 2.57 ± 2.56 and 5.08 ± 2.54 Ki67 positive cells per mm^2^, with no statistical differences. In 2-AG treated animals, the number of Ki67 positive cells of the contralateral and ipsilateral hippocampi was 13.8 ± 2.5 and 16.03 ± 16.2 Ki67 positive cells per mm^2^, respectively, with no significant differences between them.

When comparing sham and HI experimental groups, HI reduced the number of Ki67 positive cells in the contralateral hippocampus (*p* < 0.05), an effect also observed for the ipsilateral one but without statistical significance. 2AG-treated animals showed higher Ki67+ cell counts vs. sham in both contralateral (*p* < 0.001) and ipsilateral (*p* < 0.01) hippocampi. As shown before for the SGZ, Ki67 positive cell counts in animals treated with 2-AG turned out to be significantly higher both in the contralateral (*p* < 0.001) and the ipsilateral (*p* < 0.01) hippocampi when compared to non-treated HI animals. The results appear in Figure 5B.

### 2.6. DCX Cell Counts at SGZ

**Figure 6:**
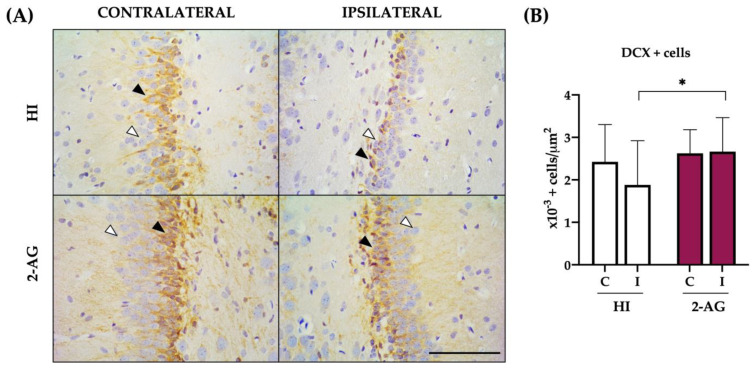
The presence of neuroblasts is more notable in 2-AG-treated animals than in non-treated HI ones at P14. (**A**) Representative microphotographs of DCX positive cells (black arrowhead) in the contralateral and ipsilateral SGZs of the hippocampus in both experimental groups. Positive cells appear stained in brown, as marked with the black arrowhead in the upper left image. Negative cells showed no brown staining, as marked with the white arrowhead in the upper left image. Images were taken at 40× magnification (Scale bar: 100 μm). (**B**) A reduced number of DCX+ cells was found in the ipsilateral SGZ of non-treated HI animals compared to the contralateral one, even though differences were not statistically significant. In 2-AG treated animals, no differences were found between the contralateral and ipsilateral SGZs. However, when comparing the ipsilateral SGZ of both experimental groups, 2-AG-treated animals showed significantly higher values of DCX+ cells (* *p* < 0.05) in comparison with non-treated HI rat pups.

At P14, the contralateral SGZ of HI animals showed 2.4 ± 0.8 × 10^−3^ DCX (a marker of neuroblasts) positive cells per μm^2^, whereas the ipsilateral one revealed lower DCX+ cell counts (1.8 ± 1.0 × 10^−3^ per μm^2^), with no significant differences between hemispheres (Figure 6B).

In 2-AG treated rats, we observed similar DCX positive cell values in both hemispheres: the contralateral SGZ showed 2.6 ± 0.5 × 10^−3^ and the ipsilateral one 2.6 ± 0.8 × 10^−3^ DCX positive cells per μm^2^, with no significant differences between hemispheres.

When comparing non-treated HI and 2-AG-treated groups, SGZ’s DCX cell counts from ipsilateral hemispheres were higher (*p* < 0.05) in animals that received the endocannabinoid 2-AG.

### 2.7. Evaluation of Hemispheric and Hippocampal Injury at P90

**Figure 7:**
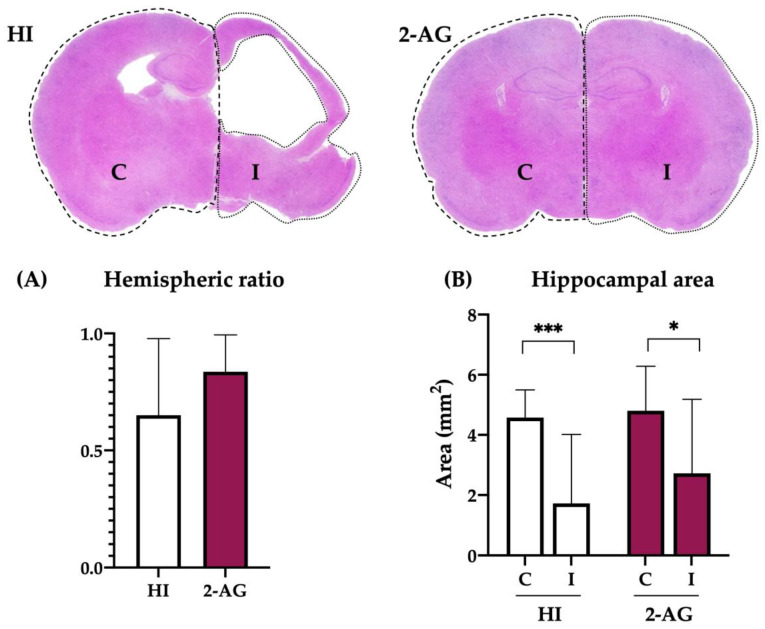
Long-term effect of 2-AG administration in hemispheric ratio values and hippocampal areas at P90. (**A**) The hemispheric ratios of HI animals were lower than those from 2-AG-treated pups, although differences were not statistically significant. (**B**) In the hippocampal area study, contralateral and ipsilateral sides showed significant differences within each group (HI *** *p* < 0.001; 2-AG * *p* < 0.05), but inter-group comparison showed no significant differences.

Long-term studies (evaluated at P90) also revealed fewer signs of infarction in the ipsilateral hemispheres of 2-AG-treated animals than in non-treated HI animals, as the hemispheric ratios obtained were 0.84 ± 0.16 and 0.65 ± 0.33 respectively (Figure 7A). However, such differences were not statistically significant (*p* = 0.19). 

We then evaluated the hippocampal injury on the same animals by measuring the hippocampal areas. At P90, ipsilateral hippocampi showed substantial injury in non-treated HI animals, revealing smaller areas than the contralateral ones (*p* < 0.001; contralateral: 4.58 vs. ipsilateral: 1.72 mm^2^). Similarly, the hippocampal areas of 2-AG-treated animals were also smaller on the ipsilateral hemisphere (*p* < 0.05; contralateral: 4.80 vs. ipsilateral: 2.73 mm^2^). However, when comparing the ipsilateral hippocampi of both groups, no differences were found, despite 2-AG-treated animals appearing to have slightly bigger hippocampi (*p* = 0.3; HI: 1.72 vs. 2-AG: 2.73 mm^2^). These results are shown in Figure 7B.

### 2.8. Neuropathological Score at P90

As we did with the P14 samples, we further assessed the global and regional neuroprotective effect of 2-AG treatment in P90 animals using the neuropathological score, a more accurate tool than hemispheric/hippocampal area evaluation. 

**Figure 8:**
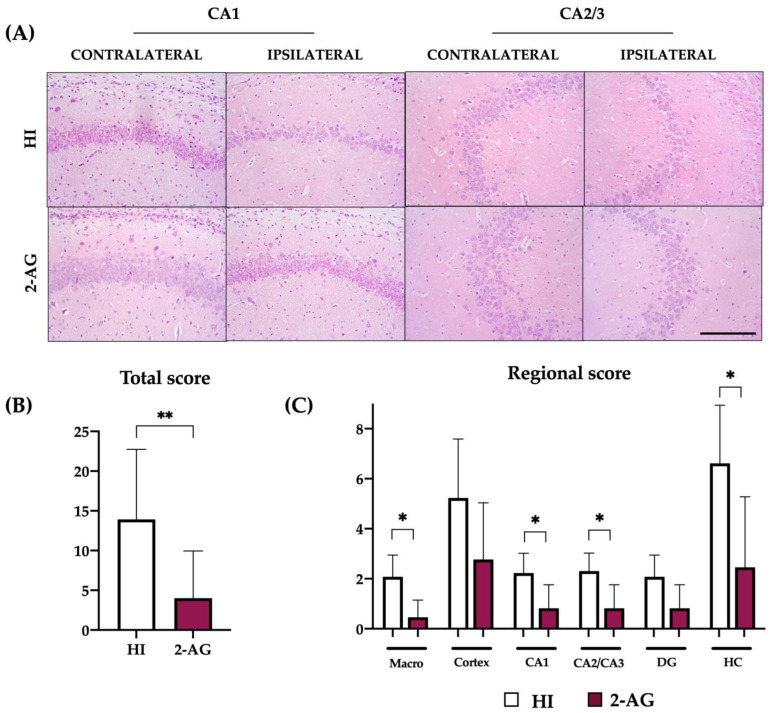
Treating animals with 2-AG resulted in lower global and regional neuropathological score values at P90. (**A**) Representative microphotographs of the contralateral and ipsilateral CA1 and CA2/3 regions of non-treated HI and 2-AG-treated animals. All images were taken at 20× magnification (Scale bar: 100 μm). (**B**) Global neuropathological score revealed significant (** *p* < 0.01) reduced damage in 2-AG-treated adult rats (P90). (**C**) Regional neuropathological scores were performed macroscopically (macro), in the cortex, in the CA1, CA2/3, and dentate gyrus (DG) areas of the hippocampus, and in the whole hippocampus (HC). Scores were higher in non-treated HI animals than in 2-AG-treated animals in all regions, but differences were only significant macroscopically, in the CA1 and CA2/3 regions and in the whole hippocampus (* *p* < 0.05).

The global score (Figure 8B) of HI animals was 13.92, whereas animals treated with 2-AG showed a significantly lower (*p* < 0.01) neuropathological score value of 4.00. When comparing the scores regionally, we found that pups receiving 2-AG showed lower values in the macroscopical injury (*p* < 0.05; HI: 2.08 vs. 2-AG: 0.46), CA1 (*p* < 0.05; HI: 2.23 vs. 2-AG: 0.82), CA2/CA3 (*p* < 0.05; HI: 2.30 vs. 2-AG: 0.82) and whole hippocampus (*p* < 0.05; HI: 6.61 vs. 2-AG: 2.41), as shown in Figure 8C. 

### 2.9. Cellularity of the DG at P90

As we observed changes in the patterns of some neurogenesis markers at P14, we assessed if these changes were reflected in an increase in cellularity at P90. 

**Figure 9:**
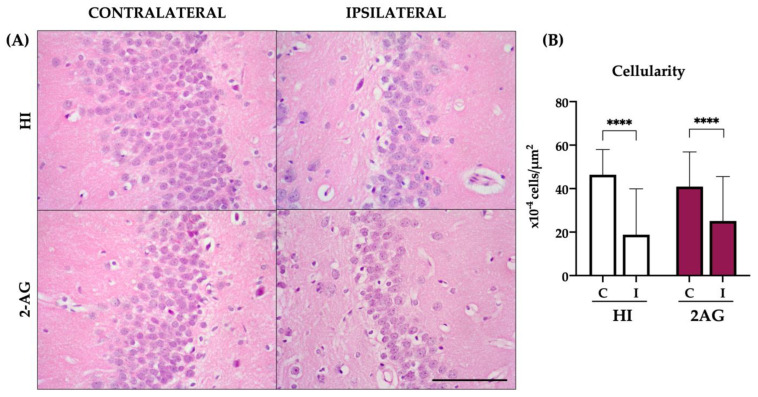
Non-treated and 2-AG-treated HI animals did not show differences in cellularity in the ipsilateral DG of the hippocampus at P90. (**A**) Representative microphotographs of the DG of the hippocampus of non-treated HI and 2-AG-treated animals. Images were taken at 40× magnification (Scale bar: 100 μm) (**B**) The ipsilateral DG of the non-treated HI group (HI) showed reduced cellularity compared to the contralateral DG (**** *p* < 0.0001). Similarly, the ipsilateral DG of the 2-AG treated group showed reduced cellularity compared to the contralateral DG (**** *p* < 0.0001). When comparing the ipsilateral DGs of the two experimental groups, no significant differences were found.

In non-treated HI animals, DG cell counts revealed reduced cellularity of the ipsilateral DG when compared to the contralateral one (*p* < 0.0001). Contralateral DGs of HI animals showed 46 ± 11 × 10^−4^ cells per μm^2^ while ipsilateral DGs showed 19 ± 21 × 10^−4^ cells per μm^2^. Similarly, the ipsilateral DG of 2-AG-treated animals showed lower cellularity than the contralateral one (*p* < 0.0001). In 2-AG-treated animals, the contralateral DG showed 41 ± 16 × 10^−4^ cells per μm^2^ and the ipsilateral ones 23 ± 20 × 10^−4^. However, no significant differences were found when comparing the ipsilateral DGs of the two groups (Figure 9). 

## 3. Discussion

In the present work, we evaluated the neuroprotective and/or neurogenic effects of the endocannabinoid 2-AG at medium- (P14) and long-term (P90) after HI in neonatal rats, showing that 2-AG treatment reduced global and regional brain injury, increased SGZ’s cell proliferation and augmented the number of neuroblasts in the DG at P14, an effect followed by global and local protection at P90.

The possibility of tissue repair in the developing brain by enhancing the proliferative potential of the neurogenic niches is currently under active investigation [26,27,28]. The modulation of the ECS to activate the neurogenic response after adult ischemia has been postulated [29,30], so we wondered if the administration of the endocannabinoid 2-AG could generate a neuroprotective and/or neuroregenerative response after neonatal HI. 

The developing brain presents several characteristics that make it especially vulnerable to perinatal HI [31], which in turn may affect the whole body of newborns [2]. In our group, we previously showed reduced brain infarct after 2-AG administration [9]; however, that work only assessed brain infarct (without a neuropathological score, a more accurate tool to assess brain damage) in the medium term (at P14), and the present work evaluates the effect of the endocannabinoid in the long term too (at P90), discussed later. Here, animals receiving 2-AG showed higher body and brain weight, higher hemispheric and hippocampal ipsilateral areas, and lower neuropathological score values at P14, all suggesting a neuroprotective effect of the treatment. These results are in line with those previously described after administering the exogenous cannabinoid WIN55,212-2 [32], an agonist of both CB1 and CB2 receptors such as 2-AG, or by inhibiting the degradation of the endocannabinoid 2-AG [12]. 

We next focused on the hippocampus, a brain area known to be essential for the acquisition and retention of spatial memory tasks, being also the location of the SGZ neurogenic niche in the DG. The hippocampus is involved in processes that are usually disrupted after deleterious events such as HI, in which brain tissue loss is one of its main characteristics [33]. Hippocampal regional vulnerability to cerebral HI during development has been described, with the DG usually being more resistant to HI, whereas the CA1 region is particularly vulnerable to hypoxia at P7 [34]. Here, we also showed this regional vulnerability of CA1 after HI, with a neuropathological score of 1.57 (max. 3) and 11/14 animals with observable infarcts. The hippocampal damage caused by this model of neonatal HI [9,35] was reverted after 2-AG treatment, with reduced infarct and lower neuropathological score values at P14 for both the whole hippocampus and CA2/3 and CA1 regions. 

Neuroprotection of hippocampal cells due to the activation of the ECS has been previously observed in vitro [36,37] and in vivo [32] in neonatal rats. We also found higher cell counts in the ipsilateral (damaged) DG of the hippocampus from endocannabinoid-treated pups seven days after HI, thus suggesting that 2-AG administration may modulate its local response to the injury. Previous works from our group using the lamb model of HI also revealed higher hippocampal cell counts after the administration of synthetic cannabinoids [10,11].

The change in DG cellularity observed in our results does not prove itself that neurogenesis is happening, as enhanced neuroprotection may also account. 2-AG is involved in central nervous system neurogenesis (for review, [38]), so we wondered if the endocannabinoid may increase cell proliferation and neurogenesis in the SGZ after neonatal HI. 

First, we showed that HI reduced cell proliferation in the SGZ: this effect has been previously described [39,40], whereas others have shown either no differences in the number of proliferating cells between HI and sham-operated animals [41] or increased [42] total counts of new cells after HI. The treatment with 2-AG increased the number of Ki67+ proliferating cells in both the whole hippocampus and the SGZ when compared to non-treated HI animals. Similarly, Fernández-López et al., [43] observed an increase in proliferating cells in the other neurogenic niche, the subventricular zone, after ECS stimulation with the synthetic cannabinoid WIN55,212-2. Interestingly, 2AG treatment was associated with an increased proliferative cell density in the contralateral hemisphere, an effect that could be related to compensatory mechanisms from the non-damaged hippocampus, previously reported after HI and melatonin treatment [44]. We next showed more DCX+ neuroblasts in the DG of 2-AG-treated animals, thus suggesting that neurogenesis is enhanced at P14. 2-AG binds to both CB1 and CB2 receptors and, besides their role in neuroprotection, it is well known that these receptors are also involved in the control of cell survival [45], neural stem cell proliferation [46], and differentiation of neural progenitors [47], key processes in any therapy in which tissue repair is the target.

The finding that 2-AG had neuroprotective/neurogenic effects at P14 prompted us to study whether the endocannabinoid provided a long-lasting effect, so we then evaluated global and regional brain damage, together with DG cellularity, at P90.

The medium-term neuroprotective effects of 2-AG were also visible in the long term, as total and regional neuropathological scores revealed fewer signs of brain damage at P90. This is of special relevance, as any therapeutic strategy with translational potential must be tested both in medium- and long-term studies. Further, the strong neuroprotective effect of 2-AG showed here has also clinical significance, as the experiments were carried out in normothermic conditions, such as in clinical settings where therapeutic hypothermia is not routine (i.e., developing countries).

An important and potentially clinically relevant difference between the responses to HI and 2-AG treatment is the special vulnerability of the hippocampus. Here, the hippocampal area was not totally recovered, nor was DG’s cellularity, despite the treatment. As recently reported, the hippocampus was the only region not protected by therapeutic hypothermia in neonatal rats [48] and in clinics [49]. We also described this particularity of the hippocampus when administering URB602 (an inhibitor of the monoacylglycerol lipase, the enzyme responsible for 2-AG deactivation): URB602 only temporarily reduced hippocampal damage after HI [12].

In the SGZ, neural progenitors divide themselves to form neuroblasts and these neuroblasts migrate to the upper DG granule cell layer [50]. The survival of these newborn neurons is however limited, as only a subset (~30%) survive at 4 weeks post-mitosis to become mature granule cell neurons [51]. The increased number of neuroblasts observed here at P14 after 2-AG administration, with no augmented DG cellularity at P90, suggests that the critical period during the integration of the immature neurons into pre-existing functional hippocampal circuits [52,53] may still be compromised despite the treatment. In line with this, reduced long-term cell survival after neonatal HI and cannabinoid treatment was previously described [43], despite WIN55,212-2 promoted neurogenesis in the medium term. So, as much as 2-AG might favor the early stages of neurogenesis in the SGZ (with higher cell counts of proliferating cells and neuroblasts), the environmental conditions of the niche may hinder the survival of these newly generated cells in the DG of the hippocampus.

This study has some limitations. We did not evaluate the long-lasting proliferative/neurogenic capacity of the endocannabinoid 2AG. P7 is an age that is representative of a human infant born at preterm or near term (32–36 weeks) and has commonly been used in neonatal HIE studies in the literature, so P7 rat pups were used in all experiments; however, since 2015 it has now been shown that P10 is the most appropriate age at which to use rat pups [48].

In conclusion, the treatment with 2-AG after HI in neonatal rats reduced brain injury and increased SGZ’s cell proliferation and number of neuroblasts in the medium term, a neuroprotective effect followed by long-term global and local protection.

## 4. Materials and Methods

### 4.1. Hypoxia-Ischemia

All experiments were in accordance with the European Union regulations for animal research (Directive 86/609/EEC) and approved by the Animal Welfare Committee of the University of the Basque Country. Neonatal HI was induced as originally described in immature rats [54] with minor modifications. On postnatal day 1 (P1), litters of Wistar rats were randomized and reduced to 10 pups per litter. Each litter contained pups assigned to the different groups of treatment with the purpose of circumventing litter effects.

On P7, rat pups were randomized to the three experimental groups before the hypoxic-ischemic procedure: (i) Sham animals (n = 6), (ii) Hypoxic-ischemic animals, which received the same volume of vehicle (see Section 4.2 below) as the treated group (HI, n = 26; 14 for P14 studies and 12 for P90 studies); and (iii) HI + 2AG treated rat pups, which received 2 mg/kg of 2-AG in a single intraperitoneal injection immediately after the end of the hypoxic procedure (n = 27; 14 for P14 studies and 13 for P90 studies).

After randomization, rats were weighed and anesthetized with isoflurane (4.0% for induction, 1.0% for maintenance) in oxygen. Using 6-0 surgical silk, the left common carotid artery of each animal was ligated in two locations and sectioned in between, to ensure there is no blood circulation. Any pup with bleeding or respiratory failure was excluded. After the surgical procedure, animals were returned to their cages and allowed to recover and feed with their dams for 1.5–2 h. After this interval, hypoxia was induced using humidified containers maintained at 36 °C and perfused with 8% oxygen with a 92% nitrogen gas mixture. After 2 h of hypoxia, rats were returned to their biological dams until P14 or P90, when they were euthanized per protocol. Sham animals were similarly managed (anesthesia and incision) but without common carotid artery occlusion or hypoxia, serving as controls. 

### 4.2. Drug Preparation

2-Arachidonoyl glycerol (2-AG, Sigma-Aldrich Co., Ltd., Gillingham, UK) was dissolved in 2% dimethyl sulfoxide + 1% Tween80 and diluted in normal saline to a final concentration of 2 mg/kg. Sham animals received a similar injection but without 2-AG in it. 

### 4.3. Tissue Processing

On P14 or P90, animals were sacrificed via lethal injection of sodium pentobarbital before brain fixation by transcardiac perfusion with phosphate-buffered saline (PBS) followed by 4% paraformaldehyde in 0.1 M PBS. Brains were removed and, after discarding the brainstem and olfactory bulb, weighed using a high precision balance (sensitivity ± 0.001 g). Data were expressed as the whole brain tissue mass. After being immersed in the same fixative at 4 °C overnight, brain tissue blocks were prepared, dehydrated, and embedded in paraffin.

**Figure 10:**
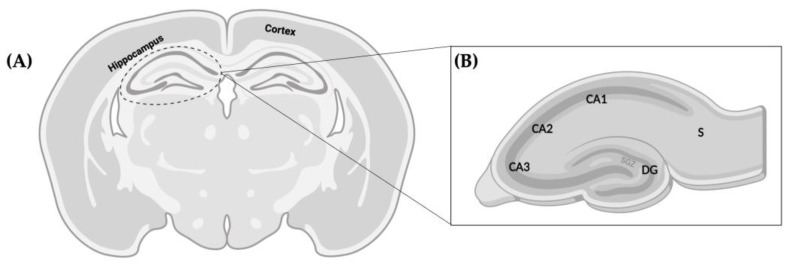
Schematic representations of the regions of the rat brain analyzed in the present work. (**A**) Representation of the coronal section at the level of mid-dorsal hippocampus and thalamus (Bregma −1.80 mm). (**B**) Magnified representation of the hippocampus, where analysis of DG cellularity and neurogenesis was performed. Relevant anatomical structures are pointed out in both images. Dentate gyrus (DG), subgranular zone (SGZ), subiculum (S).

5μm-brains sections were cut at the level of mid-dorsal hippocampus and thalamus (Bregma −1.80 mm) according to Khazipov et al., 2015 [55], where the analysis of the region of interest (Figure 10) was performed. Slides were processed for Harris’ hematoxylin and eosin (H&E) staining or for Ki67 (cell proliferation) or DCX (a marker of neuroblasts) immunohistochemistry.

### 4.4. Hemispheric Ratios

P14 and P90 H&E-stained brain slices were scanned, full-section images were obtained and measurements were performed using Fiji/ImageJ image software version 1.53k [56] by two researchers blinded to the treatment group. Ipsilateral (left, damaged) and contralateral (right, non-damaged) hemispheres were outlined manually, and the ratio of ipsi- to contralateral areas was calculated [57].

### 4.5. Hippocampal Area

Using an Olympus BX40 light microscope, 4× magnification microphotographs of the whole hippocampi were taken from each hemisphere and its area manually outlined including the CA1, CA2, CA3, dentate gyrus (DG), and subiculum using Fiji/ImageJ image software. Values were presented as square millimeters. 

### 4.6. Neuropathological Score

Brain damage was assessed at P14 and P90 on H&E-stained brain samples at the level of the mid-dorsal hippocampus and thalamus. Two researchers blinded to the experimental conditions evaluated the extent of brain damage in the parietal cortex and CA-1, CA2/3, and dentate gyrus areas of the hippocampus by using a semiquantitative neuropathological scoring system (modified from [58,59]). Brain injury was graded as follows: macroscopic damage (0–3); parietal cortex affectation (0 = no observable injury; 2 = a few small isolated groups of injured cells; 4 = several larger groups of injured cells, mild infarction; 7 = moderate confluent infarction; 9 = extensive confluent infarction); hippocampal damage in each CA-1, CA2/3 and dentate gyrus regions (0 = no observable injury; 1 = mild infarct; 2 = moderate infarct, 3 = observable cell infarction). The total score (the sum of macroscopic brain injury + region-specific injury) was graded from 0 (no damage) to 21 (maximum punctuation of damage).

### 4.7. Dentate Gyrus Cell Counts

Using P14 and P90 H&E-stained brain samples, the number of cells of the contralateral and ipsilateral DG was counted. In each sample, 8 non-overlapping microphotographs were taken at 40× magnification (4 in each hemisphere/hippocampus) and the total number of morphologically well-preserved cells was counted using Fiji/ImageJ image software. The same software was used to manually select and measure the area of DG in each photo, so values are presented as cells per μm^2^.

### 4.8. Immunohistochemistry

5-μm slices from P14 brain samples were deparaffinized, rehydrated and antigen retrieval was performed using a sodium citrate solution (10 mM sodium citrate + 0.05% Tween20 in distilled water at pH 6) where samples were boiled three times before keeping at 95–98 °C for 20 min. Once cooled at room temperature, the endogenous peroxidase was blocked with 3% H_2_O_2_ in PBS and slices were washed in PBS and incubated in a blocking solution (5% bovine serum albumin + 0.4%Triton X-100 in PBS) for 1 h. After several washes in PBS, brain sections were incubated with primary antibody to Ki67 (1:50; BD Pharmingen; #550609) or DCX (1:50; Santa Cruz Biotechnology; sc-271390) at 4 °C overnight.

The following day, slices were washed three times in PBS and incubated with secondary antibodies biotin conjugate (goat anti-mouse IgG H+L, #31800, Invitrogen, Thermo Fisher, USA) for 1 h at room temperature, followed by three washes in PBS and final incubation with horseradish peroxidase-streptavidin conjugate (1:500, 43-4323, Thermo Fisher, USA) for 30 min and posterior diaminobenzidine revealed. Finally, brain sections were counterstained with hematoxylin and mounted with DPX. Negative controls received identical treatment except for the omission of primary antibodies and showed no specific staining.

### 4.9. Ki67 Cell Counts

Ki67-positive cells were counted in the SGZ (as is the neurogenic niche of interest) and also in the whole hippocampus from both ipsilateral and contralateral hemispheres using an Olympus BX-50 light microscope. For the whole hippocampus, we first obtained a 4× photograph to measure the hippocampal area (including CA1, CA2, CA3, dentate gyrus, and subiculum) and then we counted the total Ki67 positive cells. For the SGZ, cell counts were performed on 4–5 non-overlapping microphotographs obtained at 40× magnification using Fiji/ImageJ image software version 1.53k. Values are given as Ki67 positive cells per μm^2^.

### 4.10. DCX Cell Counts

For the evaluation of DCX-positive cell counts, 3 non-overlapping microphotographs were taken at 40× magnification. In each photograph, we counted the number of DCX-expressing cells and obtained the area of the SGZ using Fiji/ImageJ image software. Values are given as DCX-positive cells per μm^2^.

### 4.11. Statistical Analyses

A two-tailed, unpaired Student’s *t*-test or one-way ANOVA (with Tukey’s multiple comparisons test) was performed for comparisons of parametric data; non-parametric data were analyzed with Mann-Whitney or Kruskal-Wallis tests (with Dunn’s multiple comparisons test). Bar graphs appear as mean ± standard deviation (SD). Statistical analysis was performed using the GraphPad Prism 8 software package (GraphPad Software, Inc., La Jolla, CA, USA). Data were considered significantly different if *p* < 0.05.

## Figures and Tables

**Table 1:** Body and brain weight of non-treated hypoxic-ischemic (HI) and 2-AG treated animals at P14.

	Sham		HI		2-AG	
	Mean	SD	Mean	SD	Mean	SD
Body weight (g)	28.33	1.33	23.65 **	3.47	27.39 ##	2.7
Brain weight (g)	1.19	0.05	0.93 ****	0.11	1.15 ###	0.08

(*) Refers to comparisons with sham animals. (#) Refers to comparisons with HI animals. ** *p* < 0.01; **** *p* < 0.0001; ## *p* < 0.01; ### *p* < 0.001.

## Data Availability

The datasets generated during and/or analysed during the current study are available from the corresponding author on reasonable request.
